# Mapping Longitudinal Dynamics of Learning Communities Dealing With Dutch Healthy Weight Approaches: An Updated Causal Loop Diagram

**DOI:** 10.1177/01632787251368438

**Published:** 2025-08-14

**Authors:** Maud J. J. ter Bogt, Kirsten E. Bevelander, Iris E. Scholte, Tessa Huttenhuis, Allia C. de Rooij, Gerard R. M. Molleman, Maria E. T. C. van den Muijsenbergh, Gerdine A. J. Fransen

**Affiliations:** 1 Primary and Community Care, Radboud University Medical Centre, Nijmegen, The Netherlands; 2 AMPHI Academic Collaborative Centre, Nijmegen, The Netherlands

**Keywords:** learning community, learning, collective action, causal loop diagram, system science, public health

## Abstract

Learning communities (LCs) stimulate the learning and cross-sectoral collaboration that are essential in multidisciplinary challenges, such as healthy weight approaches (HWAs). Previous research on two multidisciplinary LCs about HWAs devised a causal loop diagram (CLD) identifying dynamics (i.e., mechanisms) that describe the functioning of LCs during their starting phase (first 6 months after LC start). As LCs are likely to develop over project phases, this study aimed to ascertain whether and how the LC system dynamics were perceived to develop over time. Qualitative interviews conducted with the LC members at a second juncture were processed to be able to update the initial CLD. The updated diagram indeed illustrated how the multidisciplinary LCs were perceived to develop. The CLD became more extensive and consequently further explained three aspects: the complexity and interrelatedness of group dynamics, gaining insights through exchange, and conditions to execute actions. To ensure adequate group dynamics, learning, and action execution, LC stakeholders can regularly use the CLD as a blueprint to identify solutions for LC bottlenecks, such as members limitedly arranging the LC jointly. Future research is needed to investigate whether such developments are caused by different LC project phases and/or by the number of LC meetings and reflections.

## Introduction

As overweight and obesity rates keep rising (Malik et al., 2020; Ng et al., 2025; World Health Organization, 2024), the Dutch government set the goal to reduce overweight and obesity rates (Ministry of Health, Welfare and Sports, 2018, 2021, nd). Consequently, many municipalities collaborate with multiple local organizations from various sectors in applying healthy weight approaches (HWAs) (Allender et al., 2016; NWGN et al., 2021). HWAs include living environment facilities (e.g., walking paths), interventions (e.g., combined lifestyle interventions), activities (e.g., sports activities), and policies (e.g., about food offer) from an individual and an environmental perspective to enable healthy lifestyles (Allender et al., 2016; Baum et al., 2014; Huiberts et al., 2022; Kania & Kramer, 2011; Lee et al., 2016; NWGN et al., 2021). However, HWAs seem to be limitedly effective (Kobes et al., 2021; Ministry of Health, Welfare and Sports, 2024; Seidell et al., 2022), partly because overweight and obesity are complex to combat due the interplay of factors on multiple levels (i.e., individual factors, social and community networks, living and working conditions, environmental conditions) requiring interorganizational and multidisciplinary collaboration, while this complexity of obesity is not sufficiently taken into account in HWAs (Bartelink, 2019; Dahlgren & Whitehead, 1991; Nobles et al., 2021; Wilderink et al., 2022).

In 2021, five Dutch municipalities in the Gelderland region started learning communities (LCs) to create more effective HWAs by gaining insights into the actions needed to further incorporate the complexity of obesity into HWAs (ter Bogt et al., 2023). An LC is a group of stakeholders that aim to learn and collaborate by reflecting on their practices in a cooperative, exchanging, continuous, and inclusive way (Mitchell & Sackney, 2000; Stoll et al., 2006; Toole & Louis, 2002). LCs are characterized by regular LC meetings where a learning cycle is applied, such as the observe-reflect-plan-act cycle (Mann et al., 2009; ter Bogt et al., 2024) whereby LC members reflect on observations, subsequently plan actions, and then execute these actions. The literature, however, shows that it is hard to implement multidisciplinary LCs that result in learning and action (Bolam et al., 2005; Stoll et al., 2006; ter Bogt et al., 2024; TNO, 2021). More research is needed to understand the underlying patterns that determine the functioning of LCs and thereby create more effective LCs.

Previous research on understanding these underlying patterns produced a model with qualitative insights into the functioning of LCs’ system dynamics during the starting phase (first 6 months) and found four overarching themes (ter Bogt et al., 2024). The first theme of the model elucidated how group dynamics can be enhanced by members jointly organizing the LC meetings (i.e., the LC members and facilitator construct the agenda together), and why this is crucial. The second theme indicated how multidisciplinary knowledge exchange resulted in broader and deeper insights. The third theme illustrated that, if certain conditions were met, such as feeling responsible for LC actions and prerequisites like a supportive backbone, formulated actions were executed. The fourth theme explained the interrelatedness of the other three themes and emphasized the importance of involving external HWA partners in action execution and of members for setting the LC agenda (ter Bogt et al., 2024). A further understanding of these themes may help to create more effective LCs, as they explain the bottlenecks that may arise during the starting phase and how they might be overcome.

As network dynamics develop among stakeholders over the project phases (South et al., 2018), it is likely that new system dynamics will appear or that initial system dynamics will become irrelevant after years of LC meetings (Lyneis & Ford, 2007). However, little is known about system dynamic changes over time. Therefore, this is the first study to investigate the perceived dynamics of multidisciplinary LCs over time from a systems perspective. These dynamics illustrate how LC members’ behavioral changes may occur during later LC meetings. These insights will enable LC facilitators and members to intervene and continue to optimize their LCs in later LCs phases, thereby increasing the likelihood of LCs continuing to implement HWA actions adequately (ter Bogt et al., 2024). Therefore, this study aims to gain insight into whether and how the functioning of two multidisciplinary LCs’ system dynamics is perceived to develop over time, including variables and interconnectedness between variables.

## Methods

### Study Design

This study applied qualitative questionnaires and semi-structured interviews in two cases and processed them to update ter Bogt et al.’s (2024) initial causal loop diagram (CLD). The Ethical Review Board of Radboud University Medical Center assessed this study (registration number 2021-13172) and waived the requirement for a full review. We followed the ethical principles of the Declaration of Helsinki and GDPR regulations. All participants received an information letter and gave written informed consent.

### Study Setting

As part of a four-year project, two LCs were formed about HWAs (LC A and LC B). In both LCs, 11 LC meetings were organized between October 2021 and October 2024. The first three meetings took place every 6 months and lasted 4 hours, and subsequent meetings took place every three months and lasted 2.5 hours. The first two LC meetings focused on the project’s start and finding out about current HWAs, as described elsewhere (ter Bogt et al., 2024). From LC meeting 3 onwards, the observe-reflect-plan act cycle was applied as described in Table 1 (see Supplemental material 1 for more detailed programs per meeting), resulting in, among others, 609 formulated actions, as described elsewhere (ter Bogt et al., 2025a). Members additionally met in between LC meetings when desired.

**Table 1:** Overview of the General LC Meeting Agendas From LC Meeting 3 Onwards

Approx. duration*	Topic LC meeting agenda
5–10 min	Opening (welcoming everyone, agenda setting, general LC updates)
15–35 min	LC members give updates about actions on the dynamic learning agenda (Van Mierlo et al., 2010) by means of a written update form, and/or a verbal pitch, and/or plenary discussion
20–65 min	Presentation of HWA observations by the facilitator (scientific research results) and/or LC members/HWA stakeholders (own observations), followed by individual and verbal group reflection on these observations
10–45 min	Concretizing reflections and designing individual actions by participatory and creative methods (e.g., crafting, talking)
5–25 min	Plenary discussion (e.g., through art exhibition) of created plans
5–15 min	Individually writing down new action plans, resulting in a new dynamic learning agenda (Van Mierlo et al., 2010)
5–15 min	Closing (thanking everyone, agenda setting next time, opportunity for remaining questions to each other, general project updates)

*Note.* * As LC meeting 3 took 4 hours instead of 2.5 hours, topics had a longer duration for this LC meeting. Further, every LC meeting had at least one break and spontaneous moments for participants to connect and chat with one another, as was desired by LC members. Moreover, at some LC meetings, LC evaluation observations and reflections were included.

### Study Sample Procedure

HWA stakeholders were selected and invited for the LCs, as described elsewhere (ter Bogt et al., 2024). LC member iterations took place between October 2021 and October 2024, as LC members left the LC because of switching jobs (n = 18), limited returns (n = 3), limited LC relevance for own work (n = 4; of which n = 2 colleagues were replaced), personal circumstances (n = 3), too much time investment required (n = 2), and unmatched expectations/not feeling at ease (n = 1) (Supplemental material 2). Moreover, additional LC members were introduced (n = 15) or they replaced someone who had left (n = 14). The presence of LC members during LC meetings is shown in Supplemental material 3.

#### Qualitative Interviews

All stakeholders who were LC members in winter 2024 or had previously exited the LC were invited via e-mail for an interview about the LC. If they did not respond within two weeks, they received a reminder e-mail, and one week later they were phoned, resulting in 41 (89.1%) participants in total (see Table 2). Five participants – citizens (n = 3), practice professional (n = 1), policy advisor (n = 1) – did not want to participate in an interview, because of personal medical circumstances (n = 3), had participated in only one LC meeting (n = 1), or had participated in only the first two LC meetings as discussed in the 2022 interviews (n = 1) (ter Bogt et al., 2024). Therefore, their e-mails and phone call minutes with the facilitator were analyzed when available (n = 3).

**Table 2:** Exit Interview, Regular Interview, and Questionnaire Respondents per Function Category

	Exit 2023 interview (n)	Winter (January-March) 2024 interview (n)	Total questionnaire responses LC meetings 2 to 11 (n)
Municipality policy advisors	0	7	35
Municipality heath service health brokers	2	5	41
Care professional (e.g., general practitioner)	3	4	28
Practice professional (e.g., welfare worker)	6	9	56
Citizens	1	4	29
Total	12 (85.7% of former members)	29 (90.6% of current members)	189 (94.5% of present members)

#### Qualitative Questionnaire

From LC meeting 2 until 11, present LC members received an e-mail with the invitation to fill in the questionnaire. LC members who did not fill in the questionnaire within one day were sent a reminder e-mail. A last reminder was sent if the questionnaire was not filled in after the weekend, resulting in 189 (94.5%) questionnaires completed by multidisciplinary (job)function (Ter Bogt et al., 2025c) (see Table 2).

### Interview and Questionnaire Procedure

#### Qualitative Interviews

Interviews took approximately 45 minutes and were conducted via MS Teams or telephone. A semi-structured interview guide was designed based on prior monitoring tools regarding LC experiences, learning, and action (Gulikers & Oonk, 2019; ter Bogt et al., 2024; van Mierlo et al., 2010; Wagemakers et al., 2010) (Supplemental materials 4 and 5). Therefore, open-formulated questions were asked about LC evaluation, LC goals and roles, learning monitoring, acting after LC, individual learning in LC, and learning interaction with broader network, as described elsewhere (ter Bogt et al., 2024). Members had the opportunity to introduce their own topics, and follow-up questions were asked to understand causes and consequences. Exit interviews were conducted by the LC facilitator (MB), and most 2024 interviews were conducted by an independent trained researcher (IS).

#### Qualitative Questionnaire

The online evaluation questionnaire was sent at the end of the LC meeting and covered that specific LC meeting. It included open-formulated questions about good LC meeting aspects, LC meeting aspects to be improved, remark boxes, LC meeting output, next actions, and how these contributed to members’ HWA goal.

### Data Analyses

Data analyses consisted of two phases.

#### Phase 1: Thematic Analysis

Interviews were voice-recorded and transcribed ad verbatim. A thematic analysis (Braun & Clarke, 2006) was performed on both qualitative interview transcripts and questionnaire data per LC group using Atlas.ti version 9 software in two steps guided by the following research questions: (1) *How are the LC meetings perceived by the LC members?* (2) *What do LC members learn about the HWA after participating in the LC?* (3) *What underlying mechanisms play a role in LC members executing actions after LC participation?* In step 1, the final coding structure of the interview transcripts for the first 6 months, as described elsewhere (ter Bogt et al., 2024), was applied to the exit interview transcripts and open questions contained in the questionnaire for LC meetings 2–8 (MH). If new open codes emerged, these were discussed with the second coder (MB), resulting in a final coding structure for the exit interviews (MB, MH, KB). Next, the codes were merged and clustered (MB, MH) and afterwards discussed until consensus was reached within the research team (MB, MH, KB, GF). For example, the initial codes “Action group get to work”, “Don’t know subject of other working group”, “Wish successes more visible”, and “Curious whether action points are being executed” were clustered into “Visibility of success of action groups”.

In step 2, the final step 1 clusters were applied to the majority of the 2024 interview transcripts per LC group (LD, CH, MB). If new open codes emerged, these were discussed with the second coder (MB), resulting in a coding structure for the 2024 interviews (MB, KB, LD). Subsequently, codes were merged and clustered until consensus was reached within the research team (MB, KB, LD). For example, “wish more efficient LC”, “wish concrete common goal”, and “wish design LC together” all related to “LC too abstract”. Afterwards, the step 2 clusters were applied to the five remaining 2024 interview transcripts (LD, MB) and to the open questions contained in the questionnaire for LC meetings 9, 10, and 11 (AR, TH), resulting in a final clustered coding structure.

#### Phase 2: Causal Loop Diagram

The qualitative interview and questionnaire data were used to construct the qualitative CLD, which illustrates how LCs are experienced by LC members. This conceptual model shows how the clusters from phase 1 (i.e., variables) are connected (i.e., perceived causal connections) (ter Bogt et al., 2024; Waterlander et al., 2021). All connections have a positive or a negative polarity. A positive polarity illustrates that the direction of change remains the same (e.g., a perceived decreased cause is perceived to lead to a decreased perceived effect). A negative polarity illustrates that the direction of change becomes the opposite (e.g., a perceived decreased cause is perceived to lead to an increased perceived effect). These connections may ultimately balance or reinforce the original variable (i.e., create feedback loop), thereby illustrating how problems are weakened or solutions are strengthened.

The initial CLD, which was based on interview transcripts for the first 6 months, as described elsewhere (ter Bogt et al., 2024), was updated in seven steps using comparable methods (Eker & Zimmermann, 2016). First, all Phase 1 clusters of both LCs were identified as a variable (MB). Second, the LC facilitator and two other coders indicated the perceived negative or positive causal relationships between these aggregated variables (MB, MH, LD). Variables were split up or combined when perceived necessary (Kim & Andersen, 2012). For example, the variables “LC important”, “urgency LC”, and “importance of urgency LC” were all represented in “128. Urgency/importance of LC”. As the variables and their perceived causal relationships of both LCs overlapped, a single CLD was created for both LCs. Third, the perceived causal relationships were substantiated with quotes from data transcripts, and it was indicated whether the quote was an implicit or an explicit substantiation of the causal relationship (MB, MH, IS). Afterwards, 19 interview transcripts were read thoroughly to identify potential missing perceived causal relationships until no new relationships were found in three successive interview transcripts (MB, IS). Fourth, the variables and identified perceived relationships were drawn in the software program Kumu to create an updated version of the initial CLD (MB). Fifth, the CLD was critically checked and adapted where necessary by the main author (MB) and afterwards discussed until consensus was reached with the other coders (MB, MH, LD, AR, TH), two other co-authors (MB, KB, GF), and qualitative systems modelling experts (MB, MW). Small iterations were made where deemed necessary; these included sharpened variable names, excluding direct connections between variables that were indirectly connected through another variable, and splitting up or combining variables. For example, the variable “Making the LC structural” was reformulated to “Making LC components structural”, because it better matched its meaning. Sixth, the overarching themes were identified (MB, KB, GF). Lastly, 20 LC members (69.0% of interview participants winter 2024) agreed to participate in an individual member check of on average 39 minutes where they explained and gave examples of the connections that remained unclear (MB, AR, TH) (Newberry & Carhart, 2023) (Supplemental material 6). This means that every implicit connection (53 in total) was discussed in fall 2024 by some LC members until at least two participants explained in a similar way how the connection was (not) perceived, resulting in the last iterations for the final CLD.

## Results

**Figure 1:**
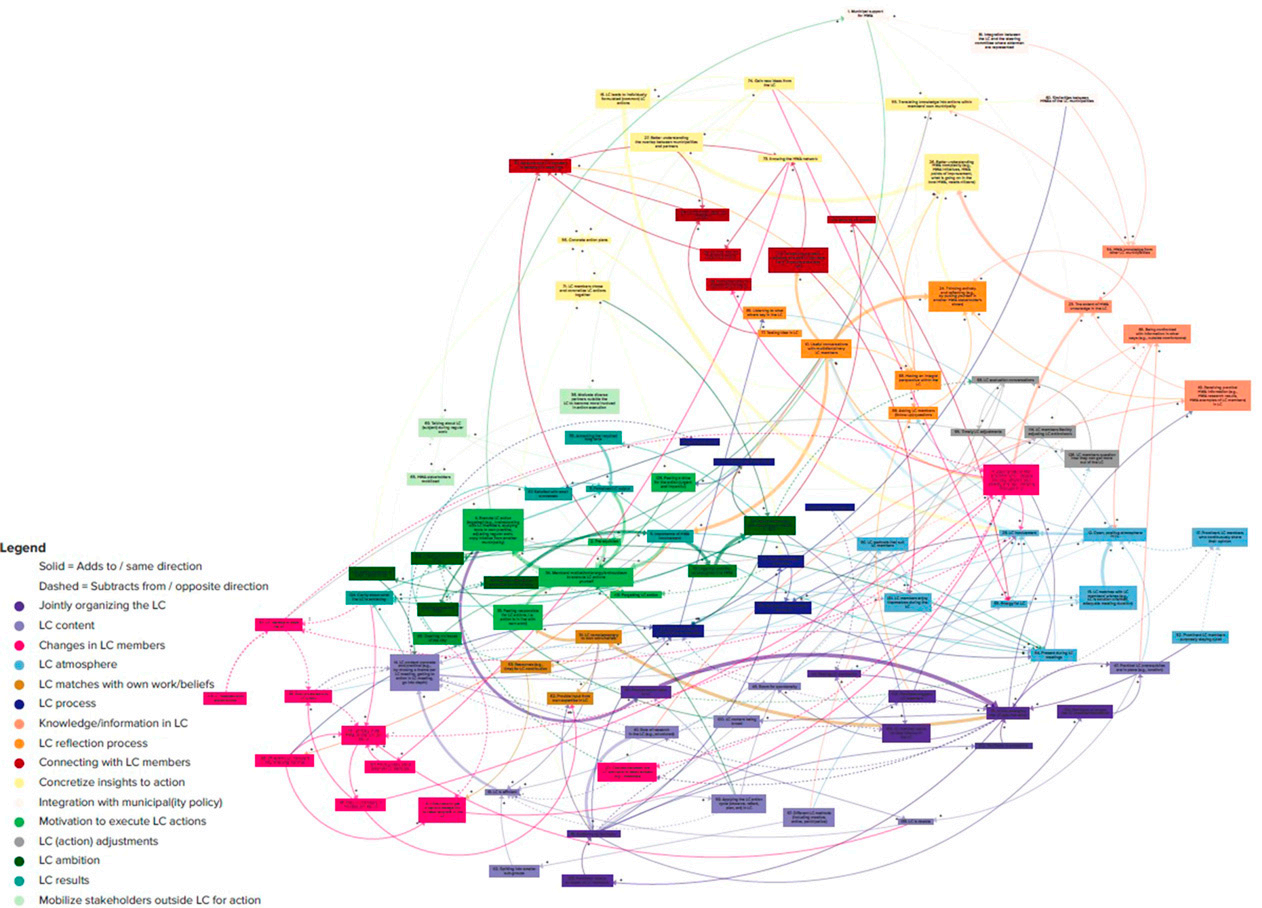
Causal Loop Diagram About Learning Communities About Dutch Healthy Weight Approaches Over Time. *Note.* Three overarching themes: (1) Group dynamics in LC (lower part of CLD, mostly blue/pink/purple colors): Jointly organizing the LC, LC content, Changes in LC members, LC atmosphere, LC matches with own work/beliefs, LC process; (2) Gaining insights through exchange in LC (upper part of CLD, mostly orange/yellow/red colors): Knowledge/information in LC, LC reflection process, Connecting with LC members, Concretize insights in action; (3) Conditions to execute actions (left middle part of CLD, mostly green colors): integration with municipal (ity) policy, motivation to execute LC actions, LC (action) adjustments, LC ambition, LC results, mobilize stakeholders outside LC for action. Arrows with a solid line indicate positive polarity, meaning that the direction of change stays the same (i.e., A increases, B increases or A decreases, B decreases). Arrows with a dotted line indicate negative polarity, meaning that the direction of change becomes the opposite (i.e., A increases, B decreases or A decreases, B increases). The color of the arrow indicates the overarching theme to which it belongs. Thick solid lines represent variables and positive connections that were already present in the CLD after 6 months; dashes (thick dotted lines) represent negative connections that were already present in the CLD after six months (ter Bogt et al., 2024). This means that all perceived LC developments over time (developments after the first 6 months) are shown in regular line thickness, while thicker lines illustrate similarities compared with the CLD after the first 6 months after the LC started. See Kumu for the more legible CLD (Ter Bogt et al., 2025b).

Overall, the updated CLD illustrated how and when LCs succeed in achieving their aim, which is collaboration (i.e., the variables LC members meeting in between LC meetings (57), LC members help one another (76)), learning (i.e., the variables better understanding HWA complexity (26), better understanding the overlap between municipalities and partners (27), gain new ideas from the LC (74)), and LC actions (i.e., the variables concrete action plans (56) and executing LC actions (4)) that potentially strengthen the HWAs. The updated CLD illustrated perceived LC developments over time by further explaining previous findings from the CLD about the starting phase (CLD T1). The updated CLD became much more extensive and included 99 variables (64 new ones compared with the initial CLD after six months) and 229 perceived connections (197 new) (see Figure 1; Ter Bogt et al., 2025b, numbers between brackets refer to the variable numbers), meaning that the new CLD further explained the previously identified overarching themes. Three aspects stood out. First, 0 variables and 18 perceived connections disappeared from the initial CLD, because the connections became indirect (as new in-between variables were added). Second, all overarching themes of the initial CLD were further explained in themes that are indicated by the colors (see Figure 1, more legible in Kumu (Ter Bogt et al., 2025b)). More specifically, group dynamics in LC was further divided into six overarching themes, gaining insights through exchange into four overarching themes, and conditions to execute actions into six overarching themes, which were interrelated. Third, we found both new reinforcing and balancing feedback loops. Figure 1 illustrates all perceived LC developments over time by regular line thickness (i.e., thick lines illustrate similarities compared with CLD T1).

Below, we describe per overarching theme and one feedback loop some new perceived LC developments over time by explaining them and illustrating an example with quotes (see Table 3). All described examples are new insights compared with CLD T1. Numbers between brackets refer to the variable numbers.

**Table 3:** The Perceived Causal Relationships Substantiated With Quotes From Data Transcripts

Number	Quote
Overarching theme: Jointly organizing the LC	
Q1	Then you have more common ownership and then you can also rotate in the one who prepares it [the learning community], for example. And that you do it much more together. (Exit interview, participant 113, lines 73–75)
Overarching theme: LC content	
Q2	Well maybe you say that because some people just have 24 forms I think. You could of course also do this in advance, right? That you say that, you know for the learning community a week in advance you send those forms online eh. Or eh and say gosh do you want to do that already then we can get started right away, so then we have that covered. (T2 interview, participant 144, lines 145–147**)**
Overarching theme: Changes in LC members	
Q3	Well, maybe that bit of concreteness. That they are still searching for how does this affect and how does it add value to my work? [And that is why they left]. (T2 interview, participant 102, lines 181–182)
Q4	[Name] I’m going to become involved in that [learning community]. But sometimes I’ve thought about partners, but then I thought, hey, then they’re going to spend so much time on that and not everyone is open to that. (T2 interview, participant 124, line 204)
Overarching theme: LC atmosphere	
Q5	I like being there [in the learning community]. Um, I get energy from it. I certainly see that last time, I also really noticed that yes, actually everyone was there and with a really good energy. And, that’s really nice to be part of that. (T2 interview, participant 127, line 29)
Overarching theme: LC matches with own work/beliefs	
Q6	I try to think along with those who are there at that moment as much as possible, a lot with my limited knowledge because it doesn’t really concern me. And maybe this is something. Maybe that is something, so I try to think along actively about that, eh but that is sometimes difficult because it is not your field of work. (T2 interview, participant 147, lines 51–52)
Overarching theme: LC process	
Q7	And what kind of success are we going to achieve with that. And there are no big successes in that. All things that come quickly also go quickly. So, I think that the path of gradualism ends up on a clay field. I think that is precisely the beauty of it. (T2 interview, participant 120, line 115)
Overarching theme: Knowledge/information in LC	
Q8	That last time yes yes, that [researcher and facilitator] had also made such a sheet. About yes, where we could pay more attention for example. Yes, I think that those kinds of things that that is something that is also really of added value, because we just don’t get that out on the table ourselves, so to speak, because you really have to do research for that. (T2 interview, participant 108, line 72)
Overarching theme: LC reflection process	
Q9	That looking integrally at the different themes and sharing signals and concerns. That that can have an effect in eh how people work. That it also creates a bit of awareness of oh, how do we deal as professionals with different themes, like I just indicated with smoking. (…) I do take with me from the learning circle. So, looking integrally at different issues. (T2 interview, participant 146, line 255)
Overarching theme: Connecting with LC members	
Q10	And, of course, I think that if you know each other it becomes even easier, but you do find each other. Because you do have certain conversations (…) but it has become a bit easier because you know people. (…) and of course it is also a networking event. You know people, you see people. Which also makes working together easier. (T2 interview, participant 162, lines 67, 110, 156–158)
Overarching theme: Concretize insights in action	
Q11	And that sometimes gives me new insights, like I hadn’t looked at it that way. And shouldn’t I adjust my policy or adjust my working method. Which also makes me connect much more with these kinds of groups. And also look at okay what can I do in the future to ensure that everyone can be helped and not those who can ask for it. (T2 interview, participant 119, line 33)
Overarching theme: Integration with municipal(ity) policy	
Q12	If partners are actively working on it, and there is energy on it, that the municipality also encourages that. That can include actively seeking budgets or something similar. (Member check, participant 112, minute 16:21) Yes, you do notice that the more things are done, the more the municipality is willing to think along with them. (Member check, participant 148, minute 55:00)
Overarching theme: Motivation to execute LC action	
Q13	What we have managed to do a bit on our own, against all resistance. With a few tough people who say we’re just going to keep going, because it has to work. And why do you do it then, you would say. Yes, it’s a kind of sacred fire that is on your heels, of we want to show that this is possible. (T2 interview, participant 128, lines 248–250)
Overarching theme: LC (action) adjustment	
Q14	Well women with the headscarves and long dresses on who want to do sports. They did not know what they wanted. Well, then you are searching together with such a group of what suits you? And then from your own frame of mind you have the idea, well easy exercises in an enclosed space (…). And when it comes down to it, it turns out that they do not like it at all. Well, then you have to start looking, what do you like? And then you start adjusting and adjusting (…). And then you adjust and then you come out with a group like that and then you have to find a gym that can do that. Well, there again you have to adjust steps every time until you finally have what you want. (Member check, participant 128, minute 1:06:47)
Q15	Well, I think it’s good that we in time eh halfway through had the conversation with each other about hey, you know, we notice that we’re running into a number of things and that’s, I think our role as [name] and I have a bit more of a signaling function in that, hey, you know, I think the setup needs to be a bit different to maintain everyone’s energy and enthusiasm and I think you responded and anticipated that very well. Eh about hey, you know, how can we better shape the project with the meetings to get more out of it? (T2 duo interview, participants 101/151, line 29)
Overarching theme: LC ambition	
Q16	We want to achieve that goal, do we agree on that? Yes, we agree on that. And then you start working together and then you are not so noncommittal, but then you know what I am steering for. (T2 interview, participant 128, line 373)
Overarching theme: LC results	
Q17	So, it is indeed in small steps of, well okay, if we find this together and do it, then we have actually already gained quite a bit. (T2 duo interview, participants 135/160, line 268)
Overarching theme: Mobilize partners outside LC for action	
Q18	I notice that when I tell and vent things about it, that people find that interesting. (…) Yes, people are enthusiastic about it so. I have some volunteers who say: yes, I want to participate in some things too. (Member check, participant 134, minute 1:08:11)
Feedback loop	
Q19	I think that it would also be good to talk about it with one another and also just to have this conversation that I am having with you now as a learning community again. What do we think, what do we think about it [the learning community]? Uh because I have the idea that I know what certain learning community participants think about it, but that is partly based on assumptions and I think that, if you talk about it, then you can also learn from one another again from okay how can we improve this for now or just continue it for the future, so to speak. (T2 interview, participant 108, line 87)
Q20	If it is more about abstract terms or relationships, then it is less practical in the sense of, okay what exactly are we going to do with it? What I see, for example, with those diagrams we start with every time. With those action points where [are] we then, on which topics we wrote to them, so to speak. Then I think yes, okay, and what does that mean, so to speak. Do we have to complete all those topics or not or what I say is the most effective. (Member check, participant 160, minute 3:37)
Q21	Hey, you know, I think the setup needs to be a bit different to maintain everyone’s energy and enthusiasm (…) Eh about hey, you know, how can we better shape the project with the meetings to get more out of it? (T2 duo interview, participants 101/151, line 29)

*Note.* Quotes of all connections are displayed in Kumu (Ter Bogt et al., 2025b).

### Group Dynamics in LC

Group dynamics expanded over time and now covered jointly arranging the LC, changes in LC members, LC atmosphere, LC matches with own work/beliefs, LC content, and LC process, which were interrelated.

#### Overarching Theme: Jointly Arranging the LC

Over time, it became clearer why members arranging the LC jointly (i.e., members and the facilitator organize the LC together, for instance by putting a topic on the LC agenda) (13) is crucial for group dynamics, as it is perceived to result in, for example, more room for spontaneity (45), and – already in line with CLD T1 – in an LC that is more complementary to members’ own work/beliefs (31), eventually contributing to more LC results. For example, when the LC is arranged more jointly (13), the facilitator listens better to the needs of LC members (103), resulting in the facilitator offering more guidance (18). When the facilitator provides more guidance (18), LC members indicated that they took less initiative, which was perceived to lead to feeling less LC ownership (44), resulting in members arranging the LC less jointly (13), the latter illustrated by Q1 (see Table 3).

#### Overarching Theme: Changes in LC Members

Over time (throughout later LC phases), changes in LC members occurred, where the appropriate member proportion (9) had a crucial contribution to group dynamics. More members were perceived to leave the LC (52) when it matched less with their expectations (113), and it was less clear what the LC was achieving (124), the latter illustrated by Q3 (see Table 3). Simultaneously, members mentioned actively inviting HWA partners to join the LC (110) when the appropriate member proportion was less than optimal (9), the group size was smaller or less stable (39), members had more ideas about potential members (107), the potential and regular members’ way of talking matched to a higher degree (109), and the LC was more doable (133), the latter illustrated by Q4 (see Table 3).

#### Overarching Theme: LC Atmosphere

The LC atmosphere makes an important contribution to LCs’ group dynamics and consists, for example, of prominent LC members purposely staying quiet (92), enjoying oneself in the LC (131), LC energy (63), LC methods that suit members (90), and being present during LC meetings (64). For example, over time, it became clear that LC energy (63) was perceived to be stimulated by having an LC that matches LC members’ wishes (15), is concrete and practical (14), educes LC support (83), and members gain new ideas from the LC (74); these were subsequently perceived to increase LC involvement (29), as illustrated by Q5 (see Table 3).

#### Overarching Theme: LC Matches with Own Work/Beliefs

The CLD also illustrates the importance of the LC being complementary to members’ own work or personal beliefs. For example, LC members perceiving that the LC is more complementary to their own work/beliefs (31) was already perceived to result in members feeling more responsible for LC actions (33) according to CLD T1. Over time, it became clear that, because of this complementarity, members subsequently were also perceived as providing, for example, more resources such as time for their LC contribution (53) and providing more input from their own expertise (62), the latter illustrated by Q6 (see Table 3).

#### Overarching Theme: LC Content

The LC content covers the LC topics and corresponding working methods during the LC. For example, CLD T1 already indicated that a more efficient LC was perceived to result in LC content that was more concrete and practical (14). In addition, over time, it became clear that LC members perceived that the LC was more efficient (19) when they split into smaller sub-groups (112), the LC was more doable (133), the LC action cycle applied after the LC starting phase was less present (59) (illustrated by Q2, see Table 3), the working methods fitted with LC members (9), and – already in line with CLD T1 – when there was less overlap between the LC and what already existed, such as meetings (20).

#### Overarching Theme: LC Process

The LC process relates to many other overarching themes and covers pushing the LC (frequency) (7), LC gradualism (135), clarity during the LC (46), LC feels mandatory (127), to be reminded of the action (117), and making LC components structural (93). For example, over time, it became clear that, when the LC was less gradual (135), members were also perceived to be less satisfied with small successes (67), as illustrated by Q7 (see Table 3).

### Gaining Insights Through Exchange in LC

Gaining insights through exchange in the LC expanded over time and now covered knowledge/information in LC, LC reflection process, connecting with LC members, and concretize insights in action, which were interrelated.

#### Overarching Theme: Knowledge/Information in LC

Over time (after the LC starting phase), more ways to exchange information arose during LC meetings and thus contributed to better understanding HWA complexity (26). For example, in addition to CLD T1, we identified over time that the extent of HWA knowledge in the LC (23) was perceived to be caused not only by the appropriate partner proportion in the LC (9), but also by HWA knowledge from other regional LC municipalities (54) and receiving practical HWA information (49), as illustrated by Q8 (see Table 3).

#### Overarching Theme: Reflection Process in LC

LC conversations were perceived to contribute to reflection and taking other perspectives, thereby further elucidating HWA complexity. For example, over time, it became clear that asking LC members more questions (88), listening better (89), and having an integral perspective within the LC (98) – illustrated by Q9 (see Table 3) – were perceived to lead to better understanding HWA complexity (26), partly through thinking more actively and reflecting (24).

#### Overarching Theme: Connecting with LC Members

Over time, it became clear how LC members connected with one another during and after LC meetings. For example, LC members who connected more with LC contacts during the LC meeting (22) also perceived that they were getting to know the HWA network better (73), and this was perceived to lead to actions that were more easily plannable (70), as illustrated by Q10 (see Table 3). This was perceived to result in LC members meeting in between LC meetings (57) (corresponding to later LC phases), which again was perceived as leading to knowing the HWA network better (73).

#### Overarching Theme: Concretize Insights in Action

Over time (after the LC starting phase), insights were concretized in LC actions, which covers for instance better understanding HWA complexity (26), knowing the HWA network (73), and choosing/concretizing LC actions together (71). For example, both more new ideas (74) and increasingly translating knowledge into actions within members’ own work or municipality (55) were perceived to result in more formulated LC actions (16), the first one illustrated by Q11 (see Table 3).

### Conditions to Execute LC Actions

Conditions to execute LC actions expanded over time and now covered integration with municipal(ity) policy, motivation to execute LC actions, LC (action) adjustments, LC ambition, mobilize stakeholders outside LC for action, and LC results, which were interrelated.

#### Overarching Theme: Integration with Municipal(ity Policy)

Integration with the municipality and its policies includes both within and between municipalities. For example, over time, it became clear that both integrating the LCs with the steering group where aldermen from the municipalities were represented (81) and executing LC actions (e.g., actions about the municipality policies) (4) were perceived to further leverage municipal support for HWAs (1), the latter illustrated by Q12 (see Table 3).

#### Overarching Theme: Motivation to Execute LC Actions

CLD T1 already illustrated that more motivation to execute LC actions was perceived to lead to more executed LC actions, which originated from various overarching themes. For example, in addition to CLD T1, we identified over time that not only the perceived output (3), prerequisites (2), the importance of HWA involvement (5), and feeling responsible for LC actions (33), but also the LC action cycle (59) applied after the LC starting phase and feeling a drive for the action (urgent and impactful) (125) were perceived to increase members’ motivation to execute actions (34), the latter illustrated by Q13 (see Table 3).

#### Overarching Theme: LC (Action) Adjustments

Flexibly adjusting the LC and LC actions became important over time. For example, regarding LC adjustments, it became clear that more members questioning how to get more out of the LC (126) was perceived to lead to more LC evaluation conversations (96) and consequently to more timely LC adjustments (95), the first illustrated by Q15 (see Table 3). Regarding LC actions, it became clear over time that, for example, members who adjusted their LC action and/or worked more flexibly (114) were perceived to execute more LC actions (4), as illustrated by Q14 (see Table 3).

#### Overarching Theme: LC Ambition

LC ambition covers what the LC wants to achieve and why. The CLD illustrates already in line with CLD T1 that feeling committed to a clear HWA goal (21) was perceived to originate for instance from seeing the overweight prevalence in the municipality (42) and feeling the urgency to strengthen the HWA together (41). In addition, over time it became clear that feeling this commitment to the goal (21) was perceived to result in, for example, working more toward the goal within the LC (129), as illustrated by Q16 (see Table 3).

#### Overarching Theme: Mobilize Stakeholders Outside LC for Action

Mobilizing HWA stakeholders who are not LC members is important for action execution. Over time, it became clear that HWA stakeholders were perceived to be mobilized to a greater extent (65) when members motivated partners outside the LC to become more involved in action execution (38) and talked more about the LC during their regular work outside the LC (69), as illustrated by Q18 (see Table 3).

#### Overarching Theme: LC Results

It is important for members to experience that the LC yields results. For example, over time, it became clear that not only accepting the required long term (35) – as already identified in CLD T1 – but also being present at more LC meetings (64) and being satisfied with small successes (67) were perceived to lead to more perceived LC output (3), the latter illustrated by Q17 (see Table 3). Like other overarching themes, the LC results theme was (in)directly interrelated with other themes regarding LC group dynamics, as well as gaining insights through exchange in LC and conditions to execute actions.

### Feedback Loops

Multiple feedback loops were identified within and throughout the overarching themes (see Figure 1). For example, over time, it became clear that, when more LC evaluation conversations took place during LC meetings (96), it was believed to result in more timely adjustments (95), as illustrated by Q19 (see Table 3). These adjustments were perceived to result in an LC that was jointly arranged to a higher degree (13). Already in line with CLD T1, this was perceived to result in a decreased role of research in the LC (40), which subsequently was perceived to result in an LC that was more concrete and practical (14), as illustrated by Q20 (see Table 3). Over time, it became clear that these more concrete and practical LCs were perceived to lead to increased LC energy (63), which subsequently was believed to result in LC members questioning to a lesser extent how they could get more out of the LCs (126). This connection is illustrated by Q21 (see Table 3). Consequently, this lesser extent of questioning was believed to result in fewer LC evaluation conversations (96), balancing the loop again.

## Discussion

The current study was the first to indicate that the system dynamics of two multidisciplinary LCs developed over time, and how, by updating the initial model. The initial CLD changed over time, providing further insights into group dynamics, learning through knowledge exchange, and conditions to execute actions for multidisciplinary LCs. For example, interrelated underlying overarching themes were discovered, such as changes in LC members (group dynamics), concretize insights in action (learning), and mobilize stakeholders outside the LC for action (actions). Therefore, LC stakeholders are recommended to apply all related overarching themes to ensure adequate group dynamics, learning, and action execution. Our findings provide several concrete solution strategies for LC bottlenecks. We discuss an overall, transcending perspective on these findings.

The importance of group dynamics, learning, and action – and the underlying variables – is in line with previous research that provided lists of important LC elements (Johannesson, 2022; Schaap & de Bruijn, 2018; Stoll et al., 2006; ter Bogt et al., 2024; TNO, 2021; Wennergren & Blossing, 2017). Therefore, we expect that this CLD may be generalized to comparable multidisciplinary LCs about HWAs. Nevertheless, LCs that are organized differently than ours (e.g., on national rather than regional level or monodisciplinary rather than multidisciplinary) may not recognize all CLD variables or connections identified in our study. Therefore, researchers studying other LCs are recommended to use our CLD as a basis and contextualize our CLD to their context when perceived necessary (ter Bogt et al., 2024). Additionally, our updated CLD illustrates that, if the LC is in another phase (e.g., starting, middle, or securing phase) (Wenger, 1998), new variables and connections are likely to appear, or existing ones to disappear. A CLD per LC phase is thus needed.

Our study showed that updating a CLD is a valuable way to study perceived LC developments over time, as the researchers’ LC observations were in line with the dynamics displayed in the CLD. Overall, the updated CLD became more extensive compared with CLD T1 by adding new variables and new perceived connections, removing perceived connections, and further explaining the original main themes – group dynamics, learning, and action – through interrelated overarching themes. These newly identified overarching themes and variables are likely to relate to changes in LC phases. For example, members could not have recognized variables such as the LC action cycle during the LC starting phase, as the action cycle was introduced only from LC meeting 3 onwards. CLD T1 provides insights into points at which to intervene on bottlenecks perceived during the first few LC meetings (i.e., the LC starting phase) (ter Bogt et al., 2024), whereas the updated CLD provides insights into intervention points during later LC meetings (i.e., the later LC phase). Consequently, qualitative researchers across all fields are recommended to investigate not only important topics or themes, but also the perceived connections between them (Gudlaugsson et al., 2022; Hairon et al., 2017; ter Bogt et al., 2024; Veldhuis et al., 2023; Waterlander et al., 2021), for instance by applying our methods to create and update a CLD. Moreover, future research is recommended to investigate the influence of repeated interviews and the number of LC meetings across the same project phase on an updated CLD. Lastly, even though the CLD illustrates the urgency of LC results and working methods for systems change, the CLD barely describes the LC results that are achieved and the LC methods that foster systems change. Therefore, more research is needed about the methods by which each LC result and each HWA system impact is achieved.

To continuously optimize LCs, LC stakeholders are recommended to look regularly at the functioning of LCs from a systems perspective. To do so, LC stakeholders, such as facilitators, can regularly use the CLD as a blueprint to identify concrete possible solution strategies (i.e., intervention points) for dealing with (potential) LC bottlenecks. For example, several variables may be targeted to (in)directly increase the extent to which the LC is jointly arranged (13); for instance, the facilitator may trigger LC members (106) to jointly arrange the LC, or make timely LC adjustments (95), or prominent LC members may purposely stay quiet (92). LC stakeholders can recognize the solution strategies that are relevant for them by identifying the overarching themes that relate to their current LC bottlenecks (current negative patterns or challenges) and which of the (in)directly related variables they are able to increase or decrease, and subsequently discuss and apply this solution strategy at an LC meeting. As actual bottlenecks differ between LCs and moments in time, possible solutions will differ too, as also suggested previously (Schaap & de Bruijn, 2018; ter Bogt et al., 2024). To further apply our CLD about LCs, researchers are recommended to quantify the CLD by investigating the strength of every connection and when variable values reach a threshold to become a bottleneck. Further, it is recommended to further develop guidelines about how the CLD can be used as a blueprint to identify solutions for LC bottlenecks.

### Strengths and Limitations

One study’s strength includes a high likelihood that all relevant experiences are adequately included in our CLD, for three reasons. First, we had a high response rate (data saturation) from both current LC members and LC members who had left the LC. Second, both repeated interviews and qualitative questionnaires at the end of LC meetings were included, meaning that both long-term reflections and short-term experiences were included. Third, member checks were performed to prevent any misunderstandings. A study limitation includes the uncertainty of whether the developments over time are caused by a different project phase, the frequency of LC meetings, or the extent of reflections. The repeated reflective interviews about the LCs or the higher number of LC meetings may have enabled members to become more conscious of their LC perceptions and identify more factors contributing to their LC’s dynamics, rather than LCs developing over time. Another limitation is that LC members were involved only in data collection and member checks rather than in the entire CLD development. We chose to do so because we applied similar methods to construct CLD T1 (ter Bogt et al., 2024), and identical methods are needed at both junctures to facilitate comparison over time. However, co-designing the CLD through methods like group model building might offer the advantage of the CLD being perceived as being produced by LC members, and this might further stimulate members to use the CLD to adjust their LCs (Littlejohns et al., 2021; Scott et al., 2016).

## Conclusion

Our study was the first to show how multidisciplinary LCs about HWAs were perceived to develop over time. The updated CLD became more extensive and consequently further explained the complexity and interrelatedness of group dynamics, gaining insights through exchange, and conditions to execute actions for LCs. To ensure adequate group dynamics, learning, and action execution, LC stakeholders can regularly use the CLD as a blueprint to identify solutions for LC bottlenecks, such as LC members limitedly arranging the LC jointly or not being sufficiently motivated to execute actions. Future research is needed to investigate whether the developments over time are caused by a different LC project phase and/or by the number of LC meetings and reflections.

## Supplemental Material

Supplemental Material - Mapping Longitudinal Dynamics of Learning Communities Dealing With Dutch Healthy Weight Approaches: An Updated Causal Loop Diagram Supplemental Material for Mapping Longitudinal Dynamics of Learning Communities Dealing With Dutch Healthy Weight Approaches: An Updated Causal Loop Diagram by Maud J. J. ter Bogt, Kirsten E. Bevelander, Iris E. Scholte, Tessa Huttenhuis, Allia C. de Rooij, Gerard R. M. Molleman, Maria E. T. C. van den Muijsenbergh, and Gerdine A. J. Fransen in The Evaluation & the Health Professions

## Data Availability

The data that support the findings of this study are available from Radboudumc but restrictions apply to the availability of these data, which were used under license for the current study and so are not publicly available. Data are, however, available from the authors upon reasonable request and with permission of Radboudumc. Therefore, data may be requested by emailing the quality team of our department of primary care (kwaliteitsteam.elg@radboudumc.nl).

## Appendix

### List of Abbreviations

HWA: Healthy Weight Approach
LC: Learning Community
CLD: Causal Loop Diagram
