# Supplementary material for: Mapping Longitudinal Dynamics of Learning Communities Dealing With Dutch Healthy Weight Approaches: An Updated Causal Loop Diagram
Source: Eval Health Prof. 2025 Aug 14;49(2):140–53. doi: 10.1177/01632787251368438 (PMC13069132; doi:10.1177/01632787251368438)
Supplement: Supplemental Material - Mapping Longitudinal Dynamics of Learning Communities Dealing With Dutch Healthy Weight Approaches: An Updated Causal Loop Diagram [file sj-pdf-1-ehp-10.1177_01632787251368438.pdf]

Supplemental material 1: Overview of LC meetings

LC meeting number	n LC A | LC B	Research input (observe)	Techniques used (methods in LC)
**3 (October 2022)**	14 | 8	Both LCs results T1 evaluation interviews [1, 2] & Results interviews low SEP citizens [3]<br><br>LC A: members' presentation	Both LCs:<br>- Presentation interviews about LC evaluation among LC members spring 2022 followed by plenary decision making on results (frequency and duration LC)<br>- Dynamic learning agenda [4] update of actions from LC 2 by means of pitches and post-its<br>- Presentation results interviews low SEP citizens<br>- Reflection on research input<br>- Planning individual and/or group actions by drawing<br>- Plenary discussion of created plans via art exhibition<br>- Listing the actions on a new dynamic learning agenda [4]<br><br>LC A:<br>- Presentation LC member about the evaluation and results of five local projects about healthy weight
**4 (January 2023)**	12 | 12	Both LCs: Results interviews LC members spring 2022: leverage point themes within HWA system [5]	Both LCs:<br>- Dynamic learning agenda [4] update of actions from LC 3 by means of pitches and post-its<br>- Presentation interviews about HWA leverage point themes among LC members spring 2022<br>- Reflection on research input<br>- Planning actions by making creations with Playmobile<br>- Plenary discussion of created plans via art exhibition<br>- Listing the actions on a new dynamic learning agenda [4]
**5 (April 2023)**	8 | 7	Both LCs: actions from previous meeting plotted on LPT scheme [6]<br><br>LC A: members' presentation about citizen participation<br><br>LC B: citizens casus	Both LCs:<br>- Short reflection on actions from previous meeting plotted on LPT scheme<br>- Dynamic learning agenda [4] update of actions from LC 4 by means of pitches and post-its; followed by plenary discussion<br><br>LC A:<br>- Presentation on behalf of 5 LC members regarding citizen participation, followed by plenary discussion<br>- Reflection and planning actions by drawing on table cover<br><br>LC B:<br>- Casus and reflection assignment about citizen participation<br>- Reflection and planning actions by making a magazine collage<br><br>Both LCs:<br>- Plenary discussion of created plans via art exhibition<br>- Listing the actions on a new dynamic learning agenda [4]

**6 (June 2023)**	9 | 7	Both LCs: results LC meeting 2, 3 and 4 evaluation questionnaire [1, 6] & actions from previous meeting plotted on LPT scheme [6]<br><br>LC A: presentation members<br><br>LC B: results HWA activity 2021 analysis & pitch two members	Both LCs:<br>- Short reflection on actions from previous meeting plotted on LPT scheme<br>- Dynamic learning agenda [4] written updates of actions from LC 5 bundled on the update wall<br><br>LC A:<br>- Repetition and deepening of citizen participation topic of LC 5 by means of individual and plenary reflection (e.g. sharing best practices, challenges, intentions)<br><br>LC B:<br>- Pitch by two LC members (1x fall prevention, 1x new communication agreements HWA)<br>- Presentation results HWA activity 2021 analysis<br><br>Both LCs:<br>- Planning actions by making drawings, writing and using DiXit cards on a table cover<br>- Plenary discussion of created plans via art exhibition<br>- Listing the actions on a new dynamic learning agenda [4]<br>- Reflection and plenary discussion on research input (results LC meeting 2, 3 and 4 evaluation questionnaire), followed by formulation of agreements for follow-up LCs
**7 (October 2023)**	8 (and 1 invitee) | 9	Both LCs: actions from previous meeting plotted on LPT scheme [6]<br><br>LC A: results HWA activity 2021 analysis & member's pitch & presentation external speaker<br><br>LC B: results prevention infrastructure questionnaire 2023 & members' pitches & reflection LC observations [1]	Both LCs:<br>- Short reflection on actions from previous meeting plotted on LPT scheme<br>- Dynamic learning agenda [4] written updates of actions from LC 6 bundled on the update wall<br><br>LC A:<br>- Pitch about activity "females who want to do sports invisibly" by LC member<br>- External speaker (invited by LC member) about needs oriented approach in practice followed by plenary reflection and discussion on citizen participation<br>- Listing the actions on a new dynamic learning agenda [4]<br>- Plan next LC meeting<br><br>LC B:<br>- Presentation results prevention infrastructure questionnaire 2023<br>- Blind spots: LC members individually pitched the blind spot he/she noticed, including why it is a blind spot, what is already present and what is missing.<br>- LC members formed groups to get more into detail among selected blind spots: Why did it not succeed yet? What and/or who is needed to succeed? What can we/I do to get started with the blind spots? Afterwards, LC members plenarily shared gained insights in the groups.

			- LC members reflected upon possible individual actions; and formulated actions on a new dynamic learning agenda [4]<br>- Vision upcoming LCs: how do we want to proceed? The idea of one health broker, policy advisor and the facilitator are pitched and reflected upon. Further, ideas for next LC member are created.
**8 (January 2024)**	12 | 7 (and 5 invitees)	Both LCs: actions from previous meeting plotted on LPT scheme [6]<br><br>LC A: Succes factors perceived by LC members [8] & members' pitches<br><br>LC B: leverage point themes citizens research [3] & results health monitor municipal health service	Both LCs:<br>- Short reflection on Both LCs: actions from previous meeting plotted on LPT scheme<br>- Dynamic learning agenda [4] written updates of actions from LC 7 bundled on the update wall<br><br>LC A:<br>- Two pitches (from LC members) (1x about communication, 1x about an intervention)<br>- Presentation success factors (as determined by LC members based on LC meeting 7)<br>- Organisational setup: "Healthy/vital citizens" was written on a paper that was put on the ground in the middle of the room. Three questions were asked; and LC members were invited to stand close to the paper if they felt much connected to the question, and further away from the paper if they felt less connected. To what extent do you feel connected to this subject? To what extent do you contribute to achieving this goal? To what extent do we contribute together to achieving this goal? After every question, a plenary discussion followed. Next, LC members were asked to write down what they do on their spot for the goal healthy/vital citizens.<br>- Groups per municipality were created to reflect and create plans: How can we (per municipality) make the HWA stronger? What are my missing pieces? Why do others already succeed?<br><br>LC B:<br>- Reflection on what leverage point themes actions were performed and on what leverage point themes no actions were performed<br>- Presentation leverage point themes citizens research<br>- Presentation main results health monitor municipal health service: mental health and young adults<br>- Several external partners regarding mental health introduced themselves. Subsequently, LC members asked questions to them.<br>- Gain insights into facilitators and barriers of mental health in two groups: youth and adults (based on writing post-its and clustering those post-its). Next, LC members were invited to reflect on: Which facilitators can you implement? Which barriers can you take away?<br>- Individual reflection on what barriers, facilitators and example you want to implement in your own work.

			Both LCs:<br>- Listing the actions on a new dynamic learning agenda [4]<br>- Plan next LC meeting<br>- Results stirring group meeting November 2023 (only via mail due to time constraints)
**9 (April 2024)**	11 | 8 (and 1 invitee)	Both LCs: results exit interviews LC members [1,9] & actions from previous meeting plotted on LPT scheme [6]<br>& members' pitches/<br><br>LC B: presentation external speaker	Both LCs:<br>- Dynamic learning agenda [4] written updates of actions from LC meeting 8<br>- Presentation results exit interviews LC members & plenary discussion<br>- Project update and reflection<br>- Reflection on what leverage point themes actions and systems levels were performed and on what leverage point themes and systems levels no actions were performed<br>- Pitches by LC members about different initiatives (LC A: 2 pitches, LC B: 1 pitch)<br><br>LC A:<br>- Exchanging about connecting within the HWA in two groups in three rounds: 1) Connecting with whom?; 2) What helps to connect?; 3) What helps to bring this example to another situation? LC members stood in a circle (indicated by a rope) and were invited to stand within the circle as long as they were talking about their own sphere of influence, and were invited to stand outside the circle when they were talking outside their own sphere of influence).<br><br>LC B:<br>- Spontaneous brainstorm about bottleneck experiences by one LC member<br>- Presentation invitee from municipality about citizen participation.<br>- Reflection by making a time line per municipality. Both municipality groups chose a topic among which they all needed one another. The time line included: 1) Situation now & what do you want to strengthen?, 2) Needed on short and long term; 3) How can you realise this together? Next, common actions were formulated.<br><br>Both LCs:<br>- Listing the actions on a new dynamic learning agenda [4]<br>- Plan next LC meeting including collaboration decisions (e.g., WhatsApp group, action updates prior LC meeting)<br>- Announcement: role facilitator will become smaller during future LC meetings
**10 (June 2024)**	10 | 8	Both LCs: actions from previous meeting	Both LCs:<br>- Dynamic learning agenda [4] written updates of actions from LC meeting 9

		plotted on LPT scheme [6]<br><br>LC A: leverage point themes citizens research [3] & all LC meeting actions plotted on leverage point themes [6] & preliminary T2 LC member interview results [1, 9]<br><br>LC B: pitch facilitator about blind spots & members' pitches	LC A:<br>- Reflection on what leverage point themes actions were (not) performed during LC meeting 9 and all LC meetings up to now & brainstorm in groups about how the leverage point themes that were not targeted in any LC action before could be incorporated<br>- Presentation leverage point themes citizens research & Reflection by making a time line per municipality. Both municipality groups chose a topic among which they all needed one another. The time line included: 1) Situation now & what do you want to strengthen?, 2) Needed on short and long term; 3) How can you realise this together? Next, common actions were formulated.<br>- Formulating and listing the actions on a new dynamic learning agenda [4]<br>- Presentation preliminary results T2 interviews LC members & subsequent reflection by individually writing down: 1) what do you want to achieve with the LCs? 2) What and whom do you need for that? Followed by plenary discussion<br><br>LC B:<br>- Pitch about on what leverage point themes actions were (not) performed during LC meeting 9<br>- Pitch about the previously discovered blind spots<br>- Six LC members held a pitch about a theme, dilemma, question, fantastic failure, or precious example from their own HWA work. LC members wrote down ideas, questions, and/or any comparable things they were already working on. After the pitches, LC members dynamically exchanged insights.<br>- Formulating and listing the actions on a new dynamic learning agenda [4]<br><br>Both LCs:<br>- Plan next LC meeting including collaboration decisions for LC B (e.g., action updates prior LC meeting)
**11 (October 2024)**	10 (and 1 invitee) | 11	Both LCs: actions from previous meeting plotted on LPT scheme [6] & LC questionnaire over time results [1, 7]<br><br>LC A: member's pitch	Both LCs:<br>- Dynamic learning agenda [4] written updates of actions from LC meeting 10<br>- Discussing actions plotted on leverage point themes<br>- Discussing results LC questionnaire over time: Recognisable? What does it mean?<br><br>LC A:<br>- Pitch about inventory healthy weight approach (by stakeholders outside LC)<br>- Plenarily discussing bottom-up relevant topics, such as including citizens bottom-up, lacking municipality plan, Fitcoins activity, neighbourhood sport coaches embedment within municipality, platform consisting of all HWA activities

		LC B: preliminary T2 LC member interview results [1, 9] & all LC actions until now plotted on LPT scheme [6] & members' pitches	- Plenarily formulating and listing the actions on a new dynamic learning agenda [4] (plenarily discussed, resulting in four actions for LC member groups)<br><br>LC B:<br>- Presentation preliminary results T2 interviews LC members & subsequent reflection by individually writing down: 1) what do you want to achieve with the LCs? 2) What and whom do you need for that? Followed by plenary LC evaluation discussion<br>- Three LC members held a pitch about a theme, dilemma, question, fantastic failure, or precious example from their own HWA work regarding citizen participation. LC members wrote down ideas, questions, and/or any comparable things they were already working on. After the pitches, LC members dynamically exchanged insights.<br><br>Both LCs:<br>- Plan next LC meeting agenda including collaboration decisions (e.g., action updates prior LC meeting, LC meeting frequency in 2025, no action forms)

The content of LC meeting number 1 and 2 are described elsewhere [2].

References

1. Ter Bogt MJJ, Bevelander KE, Molleman GRM, van den Muijsenbergh METC, Fransen GAJ. Verdiepende methoden voor het optimaliseren van leernetwerken: ontwikkel- en toepassingservaringen. Submitted.
2. ter Bogt MJJ, Bevelander KE, Kramer EAH. et al. Mapping the dynamics of learning communities about Dutch healthy weight approaches: a causal loop diagram. Arch Public Health 2024; 82: 238. https://doi.org/10.1186/s13690-024-01468-1
3. ter Bogt MJJ, Te Riele YZ, Kooijman PGC. et al. Citizens' perspectives on healthy weight approaches in low SEP neighborhoods: a qualitative study from a systems perspective. BMC Public Health 2024; 24: 2137. https://doi.org/10.1186/s12889-024-19595-3
4. Van Mierlo, B.C., Regeer, B.; Amstel, M, et al. (2010). Reflexive Monitoring in action. A guide for monitoring system innovation projects. https://www.wur.nl/en/Publication-details.htm?publicationId=publication-way-333935373332
5. Ter Bogt MJJ, Bevelander KE, Tholen L, et al. Leverage point themes within Dutch municipalities' healthy weight approaches: A qualitative study from a systems perspective. PLoS One 2023; 18: e0287050. https://doi.org/10.1371/journal.pone.0287050

6. Ter Bogt MJJ, Bevelander KE, Molleman GRM, van den Muijsenbergh METC, Fransen GAJ. Exploring learning communities' actions and perceived impact on healthy weight approaches across Dutch municipalities. BMC Public Health. 2025. https://doi.org/10.1186/s12889-025-22072-0
7. Ter Bogt MJJ, Tobi H, van Straten CGJI et al. The multidisciplinary and participatory process to develop the Rubric for Learning Communities about healthy weight approaches. *Frontiers in Public Health*. 2025, 13, 1453197 https://doi.org/10.3389/fpubh.2025.1453197
8. Ter Bogt MJJ, Bevelander KE, Philippo MP, van Eck CGM, van der Wal MM, Molleman GRM, van den Muijsenbergh METC, Fransen GAJ. Mapping the leverage point theme dynamics of Dutch municipality healthy weight approaches: a causal loop diagram. Submitted.
9. Ter Bogt MJJ, Bevelander KE, Scholte IE, et al. Mapping longitudinal dynamics of learning communities dealing with Dutch healthy weight approaches: an updated causal loop diagram. Submitted.

Supplemental material 2: LC member iterations per group

Between LC meeting 3 and 10, 8 (LC A) to 7 (LC B) new LC members were introduced, due LC members who collectively decided that their expertise was missing in the LC, or LC members introduced these professionals themselves. During the 4-year project, 9 professionals quitted the LC B, because of too much required time investment (n = 2), limited returns (n = 3), unmatched expectations/not feeling at ease (n = 1), limited LC relevance for own work (n = 1; of which n = 1 colleague took LC participation over), and personal health circumstances (n = 2; of which n = 1 colleague took LC participation over). Further, 9 professionals quitted the LC B as they switched jobs, of which 7 successors became a LC member (and one job had no successor). Further, 4 professionals quitted LC A, because of limited LC relevance for own work (n = 3; of which n = 1 colleague took LC participation over), and personal circumstances (n = 1). Further, 9 members quitted the LC A as they switched jobs, of which 7 successors became a LC member (and 2 members were not replaced as another colleague was already a LC member).

Supplemental material 3: Presence of LC members during LC meetings

Table: Number of present LC members' (job) function per LC meeting*

[LC group A | LC group B]

LC meet-ing	Municipality policy advisors	Health brokers	Care prof. (e.g., general practitioner)	Practice prof. (e.g., welfare worker)	Citizens	Total	Quest-ionnaire respons e (n)
**1**	3 | 2	6 | 4	2 | 0	2 | 2	2 | 2	15 | 12	-
**2**	3 | 2	3 | 3	4 | 2	3 | 4	4 | 2	17 | 13	17 | 12
**3**	2 | 1	4 | 2	4 | 1	2 | 3	2 | 1	14 | 8	14 | 8
**4**	2 | 2	2 | 2	3 | 2	2 | 4	3 | 2	12 | 12	11 | 12
**5**	1 | 1	3 | 2	1 | 0	1 | 3	2 | 1	8 | 7	8 | 7
**6**	2 | 1	2 | 2	1 | 0	2 | 3	3 | 1	10 | 7	8 | 5
**7**	1 | 2	2 | 2	0 | 1	2 | 4	3 | 0	8 | 9	8 | 9
**8**	2 | 2	2 | 2	1 | 1	3 | 2	4 | 0	12 | 7	12 | 7
**9**	2 | 2	1 | 2	2 | 1	5 | 3	1 | 0	11 | 8	9 | 7
**10**	2 | 2	1 | 1	2 | 1	4 | 4	1 | 0	10 | 8	10 | 8
**11**	2 | 3	2 | 2	1 | 1	3 | 5	2 | 0	10 | 11	9 | 8

* Specific participants may differ between LC meetings, even if the number of participants within one function category stays the same.

Participants attended between 1 (9% of the LC meetings) and 11 (100%) LC meetings. When excluding the participants who attended only 1 LC meeting, participants attended on average 4.2 LC meetings, while functions (participants and their successor summed up) attended on average 5.9 LC meetings. Additionally, during various LC meetings 1 to 5 HWA stakeholders were one-time present as invitee, based on the LC meeting program and LC members desires.

Supplemental material 4: Exit interview protocol

<u>Meaning of symbols</u>
- Numbered questions: questions asked
- ☐ : Possible in-depth question if answer was incomplete
- a/b/c questions were always asked, unless the participant had already answered the question in a previous answer

Introduction text – It's great that you are involved in the learning communities. I am curious about how you experience that. Can I record the audio of this conversation?

Evaluation learning community

1. Think back to your participation in the learning communities. What thoughts did you have?
   - ☐ What did you like? What did you like less?

Learning community goals and roles

2. Where do you think the learning community is working towards?
   - ☐ Do you think this will work? Why/Why not?
   - b. What is happening to achieve this goal?

Learning monitoring in the learning community

3. How do you experience adjusting your activities in the learning community?
   - ☐ What happens to make adjustments?
   - ☐ What did you like? What did you like less?

Acting after learning community

4. How did your standard working method change in the past 1.5 years?
5. What did you do after the learning community?
   a. On a scale of 0 to 10, where 0 is strongly disagree and 10 is strongly agree, how much do you agree with the following statement: "The learning community has helped me to adapt my regular working method"?
   b. Why are you giving this rating? What was different if you gave a [+2]? What was present in the learning community that you don't give a [-2]?
      - ☐ Who or what was present in the learning community that helped you further?
      - ☐ Who or what in the learning community did you need to progress, but was absent so that you did not progress?

Effect of learning community on the healthy weight approach

6. What results has the learning community achieved?
   - ☐ In your opinion, have steps been taken to strengthen the healthy weight approach in the past [number of years that the learning community has been active] years?
      a. If not, why do you think this was not successful?
      b. If yes, which ones?
      [If someone finds it difficult to put it into words] Consider, for example,

the organization of the healthy weight approach (such as collaborations), the content of the healthy weight approach (such as interventions, activities, and policy), and what residents notice about the healthy weight approach.

Individual learning in the learning community

7. What have you gained from the learning community so far?
   - ☐ What happened in the learning community / is said by someone that gave you this insight, idea or plan?
   - ☐ What did you learn from the learning community?
     - ☐ How did you / did you not learn this?
   - ☐ What has the learning community learned as a group?
     - ☐ How did you / did you not learn this?

Learning in learning community – interaction with wider network

8. How have you involved partners who are not in the learning community?

Finalizing

9. If you dreamt, what would an ideal learning community look like?
   - ☐ What is different in today's learning communities?
   - ☐ What is the same in today's learning communities?

10. Are there other things about the learning communities that we haven't discussed?

Exit questions

11. [If the participant has still an action from the previous learning community and will no longer be present at the next learning community] After learning community meeting X, you had written down your own actions: [action]
    a. To what extent was your action successful?
       Much less result than expected
       Slightly less result than expected
       Expected result
       Slightly better results than expected
       Much better results than expected
    - ☐ What did you do differently after the previous learning community meeting?
12. [If not yet known] What is the reason for you to stop your participation in the learning community?
13. Who will be your successor? How can I get in touch with him/her?
14. May I have your telephone number / email address so that I can contact you if I have any questions?

This was the last question. I want to thank you for your time and participation in this conversation.

Supplemental material 5: Interview protocol 2024

General follow-up questions that may always be used to create more clarity, such as:

- For clarification/more information:
  - What do you mean (with that)?
  - What did that look like? How did that work?
  - Can you tell me more about that?
  - What other things did you like (less)? Is there anything else? Is anything else also important to that?
- For causalities:
  - Causes:
    - What caused that?
    - How did that happen?
    - What makes this happen now (not yet)?
    - How/Why did that happen?
  - Consequences:
    - What did that to you?
    - What causes this for you? What does it result in? What does that do to you?
  - What was that like a year ago? How is it now?
- In duo-interviews:
  - And how is that for you?

<u>Meaning of symbols</u>

- Numbered questions: questions asked
- ☐: Possible in-depth question if answer was incomplete
- a/b/c questions were always asked, unless the participant had already answered the question in a previous answer

Introduction text – It's great that you are involved in the learning communities. Eight learning communities took place. I am [name], intern at [name facilitator], and I am curious about how you experience the learning communities. Can I record the audio of this conversation?

Evaluation learning community

1. Think back to your participation in the learning communities. What thoughts did you have?
   ☐ What did you like?
   ☐ What did you like less?
   c. Is there anything else?
2. [Only among learning community members who have been absent from at least three meetings]: You have been absent a couple of times.
   [People who alternate in the learning community]: You alternate in the learning community.
   a. What did that do to you [around the learning community]?
   ☐ What determined that you were there the other times?
   [People who alternate in the learning community]: What determines who comes and when?

Learning community goals and roles

3. Where do you think the learning community is working towards?
   - ☐ [If no goal mentioned]: Why do you go to the learning community? What is the goal of the learning community for you?
   - ☐ Do you think this will work? Why/Why not?
4. What is happening in the learning community to achieve this goal?
   a. Is there something else to achieve your goal?

Consequences of adjustment/learning monitoring in the learning community

5. How do you experience adjusting your work activities in the learning community?
   - ☐ How does what we do in the learning community (not) help you for your (volunteering) work?
   - ☐ What happens to adjust your work activities?
   - ☐ What did (not) help to adjust your work activities? What did you like (less)?

Acting after learning community

6. How did your standard working method change in the past 1.5 years?
7. What did you do after the learning community?
   - ☐ [If participant did not mention an action]:
     > Do you want to give an example of an action that you wrote down during the learning community? How did the follow-up go?
     > I read on the learning community agenda that you wanted to get started with [subject action in a few words]. How did that go?
   b. Is there anything else you started doing as a result of the learning community?
8. Why did this (not) succeed?
   - ☐ Was there another action that did (not) succeed? Why was this? Do you have an example of that?
   b. Is there anything else that caused the action (not) to succeed?
9. I would like to provide you a statement. On a scale of 0 to 10, where 0 is strongly disagree and 10 is strongly agree, how much do you agree with the following statement: “The learning community has helped me to adapt my regular working method”?
   a. Why are you giving this rating? What was different if you gave a [+2]? What is present in the learning community that you don’t give a [-2]? / What makes you not score lower?
      - ☐ Who or what was present in the learning community that helped you further?
      - ☐ Who or what in the learning community did you need to progress, but was absent so that you did not progress?

Effect of learning community on the healthy weight approach

10. What results has the learning community achieved?
    a. What makes this come out/happen?
    b. Are there any other results?
    - ☐ In your opinion, have steps been taken to strengthen the healthy weight approach in the past [number of years that the learning community has been active] years?
      a. If not, why do you think this was not successful?

b. If yes, which ones?
[If someone finds it difficult to put it into words] Consider, for example, the organization of the healthy weight approach (such as collaborations), the content of the healthy weight approach (such as interventions, activities, and policy), and what residents notice about the healthy weight approach.

Individual learning in the learning community

11. What have you gained from the learning community so far?
    a. What do you learn from the learning community?
    b. Is there anything else you gained from the learning community?
    c. What has the learning community learned as a group?
    - ☐ Is there anything else?

12. What made you get this [insight, idea, or plan]?
    a. Are there other reasons?
    - ☐ What happened in the learning community/was said by someone that gave you this insight, idea, or plan?
    - ☐ Why did you (not) learn this?

Learning in learning community – interaction with wider network

13. Have you involved partners who are not in the learning community?
    a. If yes, for what?
    b. If not, what made you not do this?

Finalizing

14. If you dreamt, what would an ideal learning community look like?
    a. Is there anything else in an ideal learning community?
    - ☐ What is different in today's learning communities?
    - ☐ What is the same in today's learning communities?
15. What is needed in the upcoming learning communities to move forward?
16. Are there other things about the learning communities that we haven't discussed?
17. Based on all conversations, we are creating a map about how learning communities can work. We would like to check this card with learning community participants. Would you like that and do you have half an hour for a conversation around May/June? If that is not the case, that is of course also fine.

Exit questions [only among participants who have left the learning community]

18. [If the participant has still an action from the previous learning community and will no longer be present at the next learning community] After learning community meeting X, you had written down your own actions. I will read them one by one… [action]
    a. To what extent was your action successful?
       Much less result than expected
       Slightly less result than expected
       Expected result
       Slightly better results than expected
       Much better results than expected

- ☐ What did you do differently after the previous learning community meeting?

19. [If not yet known] What is the reason for you to stop your participation in the learning community?
20. Who will be your successor? How can I get in touch with him/her?
21. May I have your telephone number/e-mail address so that I can contact you if I have any questions?

This was the last question. I want to thank you for your time and participation in this conversation.

Supplemental material 6: Member check interview protocol

The interview protocol structure and asked questions were used throughout all interviews, but the specific connections that were asked was continuously updated based on the analysis of the interviews. Part A contained checking the overarching theme names that consisted of four variables or less. Part B contained checking all connections that only consisted of implicit quotes. The interview protocol below provides one example question for part A, and one for part B. The Dutch interview protocol is translated to English. Text in black was said, text in red illustrates an action of the interviewer, and text in grey contains supplemental information that the interviewer may use when desired by the interviewee.

Introduction text

Goal: Introduce ourselves and further explain to the interviewee the purpose and structure of the interview.

[Start voice-recording]

1) Introduce ourselves
   - Hello! How nice that you are involved in the learning community and that you made time. I am [name], I am an intern of [name]. I will have this conversation with you, [name] is listening in the background and can also ask questions.
2) Ask permission for recording
   - Are you okay with me recording the audio of this conversation? Then I can focus on listening.
3) Introduce the goal of the conversation
   - Earlier this year, you had a conversation about the learning community with [name] or intern [name]. The results of all conversations have been compiled into a map about how the learning community works. I will show you that in a moment.
   - There are still a few things we are unsure about. The purpose of this conversation is to check and complete these parts.
4) Explain conversation structure
   - The conversation consists of two parts. I will explain each part when we get there.
   - You may answer briefly.
5) Are there any questions before we start?
   - [When no] Then I will now share my screen so you can watch along.

[Share screen]

   - Can you see my screen properly?

Explain the CLD

Goal: Briefly show and explain the CLD so that the interviewee has more context on what it is about.

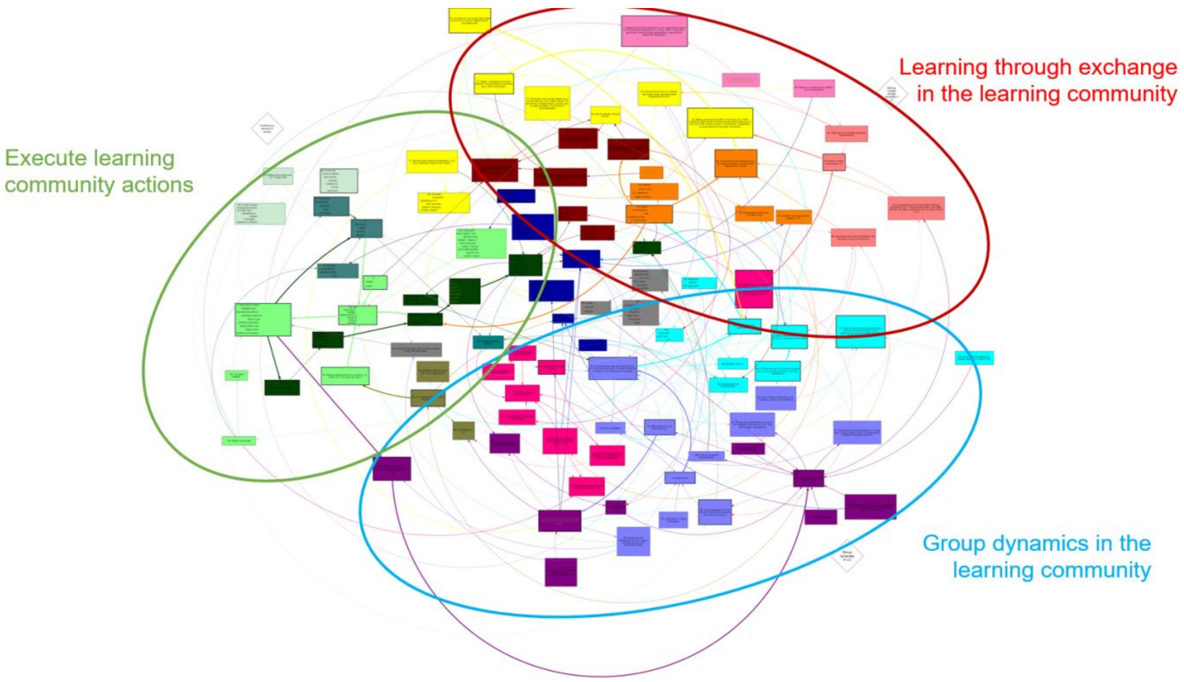

You do not have to read or understand this map. In a nutshell, this map shows how a learning community can work. Three main topics emerged from the interviews which are: 1) Group dynamics in the learning community, 2) Learning through exchange in the learning community, and 3) Execute learning community actions.

[Transition slide with Part 1]

Part A – Checking overarching themes with few variables

Goal: Check whether the overarching themes can be distinguished as a separate overarching theme.

We start with the first part. I like to present you with three statements. The question is whether you think these are important for a good learning community. You do not have to give an explanation.

- I find it important for a good learning community that… **the learning *community matches my work or beliefs.***
  Do you agree or disagree with this?
  - [When agree] Then we can go to the next statement.
  - [When disagree] Why do you not find it important? Can you explain that?

[This was the first overarching theme, continue with other overarching themes to be asked]

Part B – Checking connections

Goal: Check whether participants recognise the implicit connections and can explain them from their experiences. If they do not recognise it, find out which other cause/effect the variables have which make them increase/decrease.

This was part one of the conversation, now we go to part two.

[Slide with marked main topic, for example group dynamics in the learning community]

We are going to talk about group dynamics in the learning community. This is about why group dynamics is important in a learning community, and how this can be strengthened when the learning community is arranged together.

You get to see two topics each time. I am curious if you experience a connection between them. I would like to go through [number] connections. We will go through them one by one.

If the PowerPoint says that if one becomes more, the other also becomes more, the opposite is also true: if one becomes less, the other also becomes less.

You may answer briefly.

[Slide with the first connection]

The first few connections are about changes in learning community members.

- If more members quit the learning community, do you notice that more new members actively get invited?

  If less members quit the learning community, do you notice that less new members actively get invited?
  Example first variable: LC members quit
  Example second variable: Invite external partner, ask new people for the LC

  - [When yes] Do you have an example of this?
  - [When no] What do you not recognise?
    - *[Cause] What happens then if more/less members quit the learning community?*
    - *[Effect] What then causes more/less new members to get actively invited?*

This way, we will discuss all [number] connections. Is that clear?

[This was the first connection, continue with other connections to be asked]

Closing up the conversation

We went through the main topic Group dynamics in the learning community. Are there any other things important within this topic that we have not discussed yet?

- [When yes] *Can you give an example? / When did you notice this*?
  - [When cause not mentioned]: *What caused this?*
  - [When effect not mentioned]: *What does this lead to?*
  - [To check if you have everything] *Is there anything else important?*
- [When no] *Is there anything else you would like to say?*

Well, then we are done. Thank you very much for your time and input. We certainly got something out of it! Have a great day!

Distribution of overarching themes and connections across participants

The table below provides an overview of the distribution of the overarching themes and connections across participants. This distribution was used at the start of the member check interviews. After nine member checks, Part A was left out. After sixteen member checks, the subparts B1-B2-B3 were merged into one part B with the connections that were not yet solved.

**Part A: the overarching themes**	
**Subpart A1**	**Subpart A2**
LC matches with own work/beliefs	LC (action) adjustments
Mobilise stakeholders outside LC for action	Knowledge/information in LC
Integration with municipal(ity policy)	LC results

**Part B: connections corresponding to the overarching themes**		
**Subpart B1**	**Subpart B2**	**Subpart B3**
Group dynamics	Group dynamics	Conditions to perform actions
Jointly organising the LC	LC content	Integration with municipal(ity policy)
Changes in LC members	LC process	
LC atmosphere		
LC matches with own work/beliefs	Insights through learning	Motivation to execute LC actions
	Knowledge/information in LC	LC (action) adjustments
	LC reflection process	LC results
	Connecting with LC members	Mobilise stakeholders outside LC for action
	Concretise insights into actions	
